# A review of the influence of biogeography, riverine linkages, and marine connectivity on fish assemblages in evolving lagoons and lakes of coastal southern Africa

**DOI:** 10.1002/ece3.3266

**Published:** 2017-08-10

**Authors:** Alan K. Whitfield, Steven P. Weerts, Olaf L. F. Weyl

**Affiliations:** ^1^ South African Institute for Aquatic Biodiversity (SAIAB) Grahamstown South Africa; ^2^ Council for Scientific and Industrial Research (CSIR) Durban South Africa

**Keywords:** coastal lakes, fish composition, Holocene, sea level changes

## Abstract

The Holocene evolution of eight South African coastal lakes and lagoons is examined and related to changes in fish composition over that period. Historical and current connectivity with riverine and marine environments are the primary determinants of present‐day fish assemblages in these systems. A small and remarkably consistent group of relict estuarine species have persisted in these coastal lakes and lagoons. The loss or reduction of connectivity with the sea has impacted on the diversity of marine fishes in all eight study systems, with no marine fishes occurring in those water bodies where connectivity has been completely broken (e.g. Sibaya, Groenvlei). In systems that have retained tenuous linkages with the sea (e.g., Verlorenvlei, Wilderness lakes), elements of the marine fish assemblage have persisted, especially the presence of facultative catadromous species. Freshwater fish diversity in coastal lakes and lagoons is a function of historical and present biogeography and salinity. From a freshwater biogeography perspective, the inflowing rivers of the four temperate systems reviewed here contain three or fewer native freshwater fishes, while the subtropical lakes that are fed by river systems contain up to 40 freshwater fish species. Thus, the significantly higher fish species diversity in subtropical versus temperate coastal lakes and lagoons comes as no surprise. Fish species diversity has been increased further in some systems (e.g., Groenvlei) by alien fish introductions. However, the impacts of fish introductions and translocations have not been studied in the coastal lakes and lagoons of South Africa. In these closed systems, it is probable that predation impacts on small estuarine fishes are significant. The recent alien fish introductions is an example of the growing threats to these systems during the Anthropocene, a period when human activities have had significant negative impacts and show potential to match the changes recorded during the entire Holocene.

## 1 INTRODUCTION

Natural lakes and lagoons are rare in South Africa because the climate is generally arid (mean annual rainfall is <400 mm), and surface water flow is dominated by the Orange River which contains 85% of the surface freshwater in the country (Weyl & Cowley, 2015). Other drainages are relatively short, and typically comprise geographically isolated, short coastal rivers which descend rapidly from mountain ranges into the ocean. As a result, most natural lakes and lagoons are ephemeral. Permanent lakes are typically of estuarine origin, formed as a result of changes in sea level between the late Pleistocene and Holocene.

Transformations of estuaries within the Holocene are fairly well understood (de Lecea, Green, & Cooper, 2016). Although sea level has remained relatively constant for the last 7,000 years, estuaries are, on geological time frames, ephemeral (Schubel & Hirschberg, 1978). Typically the hydrological evolution of these systems after the early Holocene sea level rise has involved sedimentation from terrigenous sources, shallowing, reductions in volumes and associated reductions in tidal prisms and marine influence (Cooper, 2001a). In general, increasing isolation from the sea occurs, with many South African systems evolving into temporarily open/closed estuaries (Cooper, 2001b) and some becoming completely isolated from the sea (Hill, 1975).

**Figure 1:**
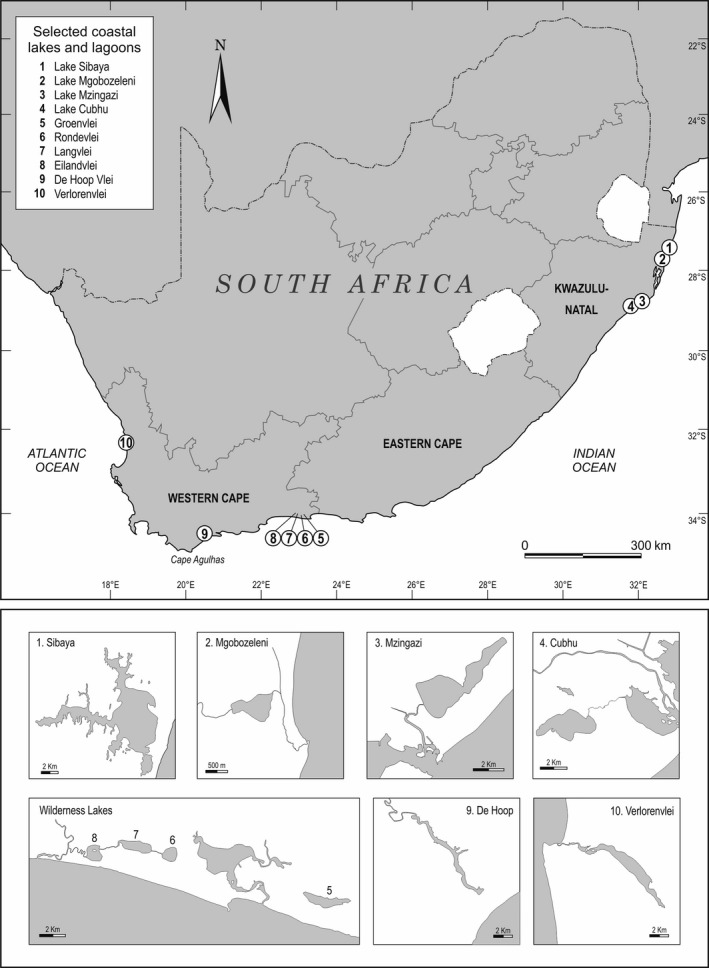
Map showing the location of coastal lakes and lagoons covered by this review

As a result of topography, estuarine and coastal lakes in South Africa are located either along the temperate south and west coast of the Western Cape Province or on the subtropical coastal plains of north eastern KwaZulu‐Natal (Figure 1). Fish communities of coastal lakes and lagoons in particular make for interesting study, as current diversity and composition represents the interplay between colonization opportunities, the degree of connectivity to marine and freshwater environments, and the compatibility of the life‐history traits of the colonizing fish faunas in alignment with the evolving physicochemical environment.

From a freshwater ecoregion perspective for example, the temperate lakes are located within the freshwater Cape Fold Ecoregion where deeply incised river valleys limit lateral connectivity between rivers which typically contain 12 or fewer native freshwater fishes (Ellender, Wasserman, Chakona, Skelton & Weyl, 2014). The subtropical lakes, on the other hand, are located in the lower Zambezi Freshwater Ecoregion where low gradient rivers, typically containing more than 40 freshwater fish species, are periodically connected during times of flood (Thieme et al., 2005). Similarly marine and estuarine fish faunas are typically richer in species composition in subtropical than in temperate estuaries (Harrison & Whitfield, 2006). As the lakes become more and more disconnected from these source populations, species with incompatible life histories (e.g., marine spawning species) disappear and are replaced by species with wide tolerances of salinity and the ability to reproduce in the now lentic environment (Allanson, Hill, Boltt, & Schultz, 1966). As a result, the fish community in each coastal lake is unique.

As is the case with many aquatic ecosystems (Global Biodiversity Outlook 4 2014), estuarine and coastal lakes are under increasing threat from multiple stressors linked to societal demands on water and the concomitant impacts of impoundment, flow reduction, and alien species introductions. These impacts are expected to be exacerbated as climate change and a growing human population imposes an ever‐stronger footprint on natural ecosystems (Jackson, Woodford, & Weyl, 2016) and are likely to be particularly severe in arid regions such as South Africa, where climate change predictions are that rainfall, and thus flows, are likely to become more erratic and less predictable (Intergovernmental Panel on Climate Change, 2014). Understanding the consequences of reduced flows, and consequently connectivity, to estuarine and coastal lakes is therefore important for their conservation.

The objective of this review was to examine the formation processes, connectivity, and changing fish species composition of eight coastal lakes/lagoons that represent varying stages of disconnection with the estuarine and marine environment. This review highlights the unique nature of these water bodies and describes the changing evolutionary trajectory of fish communities within each system as the connection with the estuarine environment is gradually lost. It also emphasizes the important role that humans are playing in recent times by introducing alien fish species into these unique coastal habitats, and the consequences of those introductions on ichthyofaunal composition.

## 2 TEMPERATE COASTAL LAKES/LAGOONS

### 2.1 Groenvlei

The coastal valleys of the southern Cape, including the Swartvlei Valley, were deeply incised during long periods of glacio‐eustatic sea level regressions that occurred between 1.5 million and 14,000 years BP (Truswell, 1977). During the last sea level regression that occurred during the Pleistocene, and resulted in a level of about 120 m below the present MSL (Ramsay & Cooper, 2002), wind‐blown sand from the drying out Agulhas Shelf region would have formed very large dune fields that provided the sediment source for the later infilling of the lower Swartvlei Estuary valley and adjacent Groenvlei arm of that valley (Martin, 1962).

The hills on the northern side of Groenvlei represent an ancient middle dune cordon 150 m high that was once a sea cliff, with the hills on the southern side of Groenvlei forming a more recent seaward cordon about 150–170 m high (Illenberger, 1996). The consolidated seaward cordon provides an aeolianite ridge that has protected the Groenvlei Lagoon, which is a depression lying between the two cordons, from being filled in by mobile marine and coastal sediments during the last 100,000 years. According to Martin (1968), this depression has been flooded by seawater on more than one occasion in the past, once about 38,000 years BP and again following the Holocene rise in sea level.

**Figure 2:**
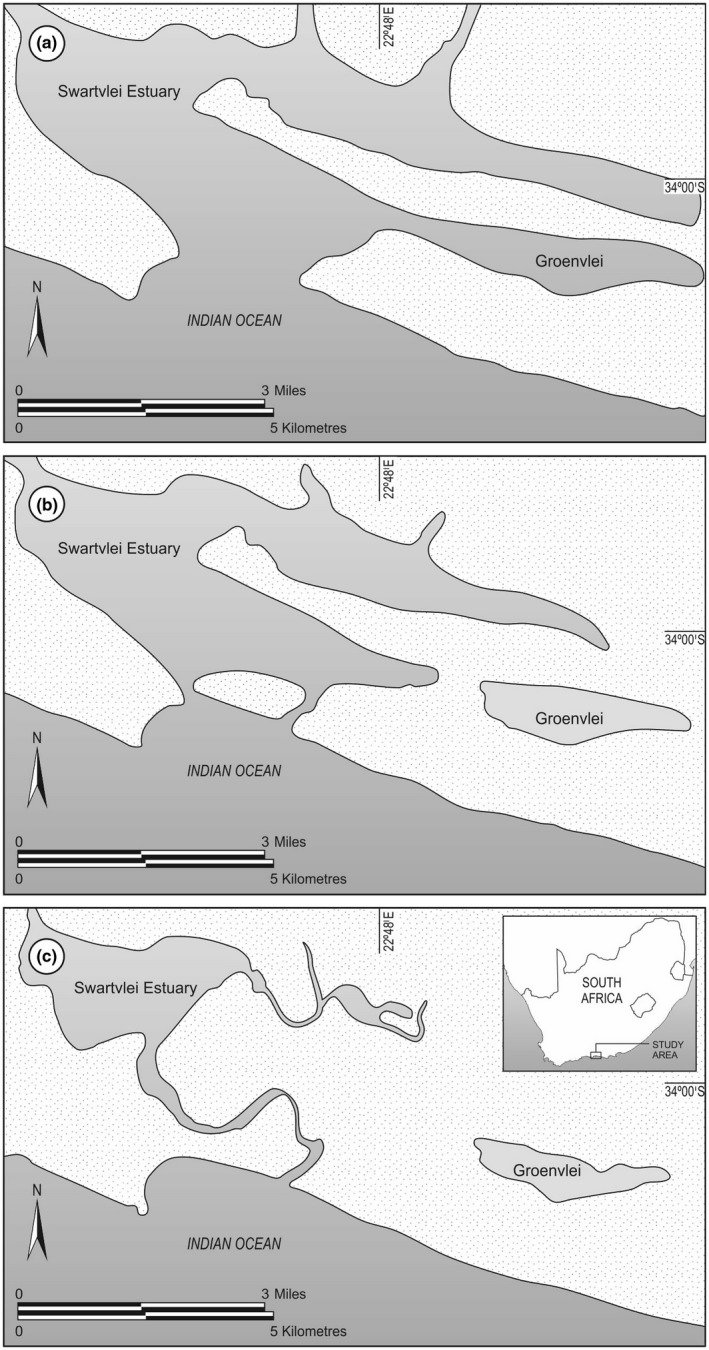
(a) Map showing the probable configuration of the link between the Swartvlei Estuary and the Groenvlei “arm” approximately 4,500 years BP. Note the very wide but shallow sandy Swartvlei estuarine embayment. (b) Loss of connectivity between the Swartvlei Estuary and Groenvlei occurred between 4,000 and 2,000 years BP and a subsequent rise in sea level about 1,500 years ago failed to reconnect the lagoon to the estuary. (c) Current map of the Swartvlei Estuary and Groenvlei

Fen borings just east of the current Groenvlei shoreline show that the earliest sediment in the area was deposited in a small freshwater lake (Martin, 1959). From about 8,000 years BP, the Groenvlei system became linked to the Swartvlei Estuary and formed an estuary “arm” or lagoon to the east of the Swartvlei system (Figure 2a). This view is supported by the presence of marine sediment immediately east of the current Groenvlei shoreline dated at 6,900 years BP and which contained a mainly marine diatom flora (Martin, 1959). The estuarine lagoon would have been deeper than the present‐day Groenvlei, especially during the mid‐Holocene sea level highstand about 4,500 BP when the sea level was 3.5 m higher than present (Figure 2; Ramsay, 1995), and the lagoon would have been fully tidal in synchrony with the main Swartvlei Estuary to the west. The estuarine conditions in Groenvlei would have persisted for at least 4,000 of the last 10,000 years (Martin, 1959).

**Figure 3:**
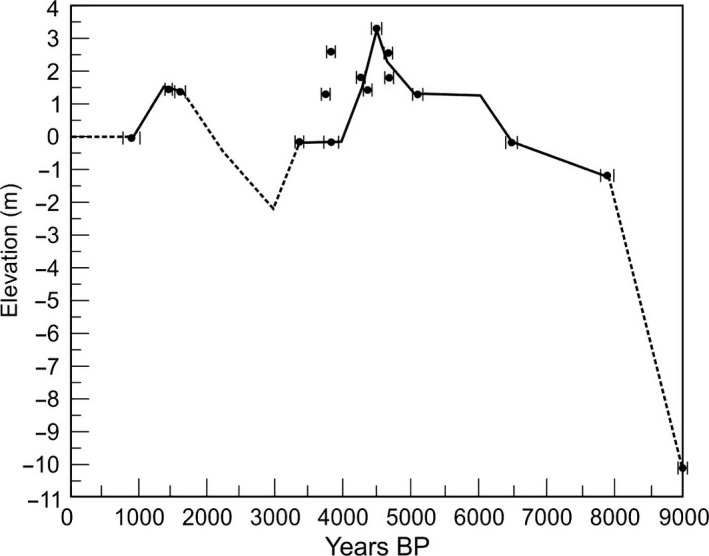
Holocene sea level changes in southern Africa (after Ramsay, 1995)

With the retreat to lower sea levels between 4,000 and 3,000 years ago (Figure 3; Ramsay, 1995), and the ingress of mobile sand dunes to the east of the Swartvlei Estuary, the link between the estuary and Groenvlei Lagoon was finally severed (Figure 2b). In the absence of any river flow from the small 25 km^2^ Groenvlei catchment to scour this sand “blockage” during the lower sea level period, these dunes would have remained in place and grown in size, finally becoming stabilized by vegetation (Martin, 1968; Figure 2c). A subsequent rise in sea level to +1.5 m about 1,600 years BP (Figure 3; Ramsay, 1995) would have been insufficient to breach the stabilized dune field that was isolating Groenvlei from the Swartvlei Estuary. This scenario is supported by Martin (1959) who presents strong evidence that freshwater conditions were again dominant in the lake about 1900 years BP, with marine or estuarine diatoms absent from the more recent Groenvlei deposits.

Physicochemical conditions in the earlier Groenvlei estuarine lagoon would have closely resembled those in the present‐day Swartvlei Estuary, with salinities ranging between 20 and 35 in the lower reaches and 15–35 in the middle reaches during the open mouth phase (Whitfield, 1988a). During the closed mouth phase, salinities in the estuary tend to decline as lower salinity water (8–12) from the Swartvlei lake enters the estuary. Salinities in Groenvlei would therefore have been predominantly polyhaline during the Swartvlei Estuary open mouth phase and mesohaline during any closed mouth phase.

The present‐day Groenvlei Lagoon is 4 km long, about 1 km wide in the middle (Figure 2c), and has a surface area of approximately 2.5 km^2^. There is no river or stream entering the system but it is fed by both direct rainfall (71.6%) and groundwater (28.4%) (Parsons, 2014). The maximum depth of the lagoon is approximately 5.5 m with most of the central areas being about 2–3 m in depth. The salinity of Groenvlei is 2–3 and retention of salts within the system is promoted by the vegetative fringe around the system (Parsons, 2014).

Based on the occurrence and distribution of macrophytes in the Swartvlei system (Howard‐Williams & Liptrot, 1980), the aquatic vegetation of the Groenvlei lagoon was probably dominated by *Ruppia cirrhosa* in the subtidal areas due to the sheltered nature of the lagoon. The eelgrass *Zostera capensis* would almost certainly have occurred in the channel leading into the Groenvlei estuarine lagoon due to its polyhaline salinity status and close proximity to the Swartvlei Estuary mouth. *Phragmites australis* would have been present in supratidal and intertidal areas where freshwater seeps occurred, and the salt marsh plants *Sarcocornia perennis*,* Salicornia meyeriana,* and *Juncus kraussii* would have dominated in more saline soils around the high water mark.

With the loss of estuarine connectivity about 3,000 years BP, the vegetation of the Groenvlei system would have changed very quickly. All *Z. capensis* in the previous intertidal and subtidal areas would have disappeared and been replaced by submerged macrophyte beds of *R. cirrhosa* while salinities remained polyhaline or mesohaline. *Potamogeton pectinatus*,* Ceratophyllum demersum,* and Charophyta would have gradually replaced the submerged *R. cirrhosa* beds as salinities moved from mesohaline toward oligohaline levels, and the littoral *P. australis*,* Typha capensis,* and *Schoenoplectus scirpoides* beds would have expanded into the lagoon under the same conditions.

Most of the typical estuarine copepod, isopod, amphipod, tanaid, polychaete, and bivalve species would have disappeared soon after severance of the estuarine connection with Swartvlei. This change is reflected in a comparison in the diet of *Atherina breviceps* and *Gilchristella aestuaria* from the Swartvlei Estuary and Groenvlei (Coetzee, 1982). The fish from Swartvlei had a number of invertebrate species in their diet that were absent from the stomach contents of the same species from Groenvlei.

### 2.2 Past fish assemblages

During the Eemian interglacial period (130,000–115,000 years BP), and prior to start of the major cycles of glaciation in the northern hemisphere between 80,000 and 20,000 years BP (Ramsay & Cooper, 2002), the sea would have completely covered the current Groenvlei valley (Illenberger, 1996), with typical coastal marine fish species occupying the area right up to the northern escarpment of the current Swartvlei lake. In the absence of any fish fossil records in this area from the Eemian, we can only speculate on the species involved.

With the retreat in sea level associated with the major glacial periods in the latter stages of the Pleistocene, the Groenvlei Valley would either have dried out or, depending on its shape and configuration, retained a wetland area between the northern and southern aeolianite ridges, probably at the site of the current Groenvlei. The fish fauna in the wetland would not have been freshwater derived as no river flowed through this very small coastal catchment. It is therefore quite possible that the Groenvlei wetland 15,000–80,000 years BP was devoid of any fish species, unless *A. breviceps* and *G. aestuaria* managed to occupy the ancient Groenvlei prior to the first steep drop in sea level about 80,000 years BP.

**Figure 4:**
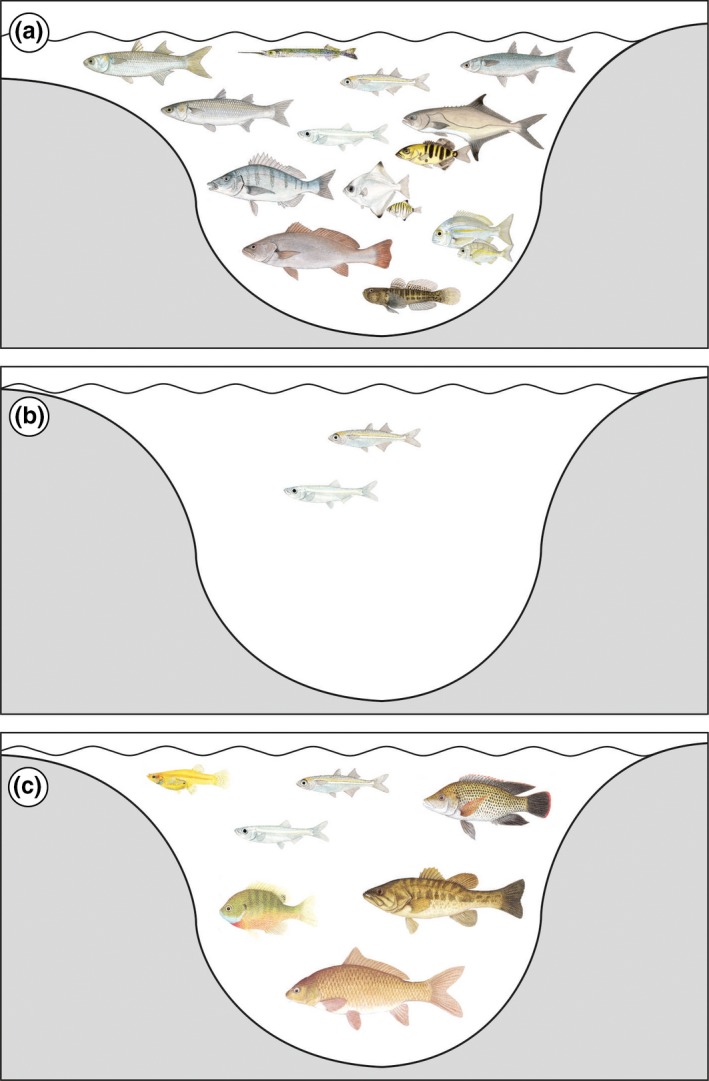
(a) Probable fish species in the Groenvlei estuarine lagoon about 5,000 years BP. (b) Fish species remaining in the Groenvlei coastal lagoon about 2,000 years BP. (c) Changes in fish species assemblage in the Groenvlei coastal lagoon during the last 100 years, driven by non‐native introductions

With the marine inundation of the Groenvlei valley approximately 7,000 years BP, a fish assemblage, probably dominated by estuary‐associated marine species, would have taken occupation of the Groenvlei arm of the Swartvlei estuarine system (Figure 4a). This fish assemblage, which would have closely resembled the current ichthyofauna of the Swartvlei Estuary (Whitfield, 1988b), is likely to have persisted until at least 3,500 years BP when sea level started to decline to −2 m below current MSL about 3,000 years BP (Figure 2; Ramsay, 1995) and would have resulted in a drying out of the connection between the Swartvlei Estuary and Groenvlei Lagoon. The trapped marine fish species would become adults in Groenvlei but would have been unable to breed within the system (Whitfield, 1999). Within 100 years of the loss of connectivity with the sea, all these marine species would have disappeared from the Groenvlei system. Only the relict estuarine spawning, *A. breviceps* and *G. aestuaria* would have persisted within the Groenvlei Lagoon from about 3,000 years BP to Present (Figure 4).

Between about 3,000 and 2,000 years BP, a mobile sand dune formed across the narrow Groenvlei valley immediately west of the current lagoon (Figure 2b), thereby cutting off any future potential link between Groenvlei Lagoon and the Swartvlei Estuary. This new dune cordon, which was then colonized by vegetation, effectively prevented the transformation of the Groenvlei coastal lagoon into an estuarine lagoon during subsequent rises in sea level. Therefore, no estuary‐associated marine fish species would have been able to enter the system during the short‐lived marine transgression about 2,000–1,500 years BP when the sea level rose to +1.5 m MSL (Figure 3; Ramsay, 1995). Thus, the Groenvlei Lagoon remained isolated and its fish fauna comprised only two species (*A. breviceps* and *G. aestuaria*) between about 3,000 and 100 years BP.

### 2.3 Present fish assemblages

Only *G. aestuaria* was sampled in the early 1900s in Groenvlei, leading to the conclusion by certain freshwater ichthyologists and fishery management authorities that the introduction of non‐native recreational fish species would be appropriate (Harrison, 1952). Subsequent intensive fish sampling of Groenvlei showed that a second indigenous fish species, *A. breviceps*, was also abundant in the lagoon and that both *A. breviceps* and *G. aestuaria* were recently genetically differentiated from other estuarine populations of these species in South Africa to such an extent that the Groenvlei stocks deserve special conservation attention (Phair, Barendse, Smith, & von der Heyden, 2015).

Unfortunately formal fish stocking of various inland waters with non‐native freshwater fish species was encouraged by provincial conservation departments in South Africa during the early and mid‐1900s, and 15 largemouth bass *Micropterus salmoides* fingerlings were introduced into Groenvlei in 1934 (Jubb, 1973). This successful introduction of *M. salmoides*, and the perceived vacant ecological niches available for other non‐native fish species, provided momentum for the introduction of additional alien fishes. Bluegill *Lepomis macrochirus*, Mozambique tilapia *Oreochromis mossambicus*, and western mosquitofish *Gambusia affinis* were introduced in the 1940s. Following on from these government‐approved fish introductions to the system, an illegal introduction of the common carp, *Cyprinus carpio*, occurred in about 1990 (Olds, Smith, Weyl, & Russell, 2011). All five non‐native species of fish (Figure 4c) have successfully established in Groenvlei (Weyl, Ellender, Wassermann, & Woodford, 2015) but their impact on the ecology of the system has not yet been determined.

### 2.4 Verlorenvlei

This system (32°20′S; 18°24′E) is in the final stages of transition from an estuarine lake into a coastal lake. The vlei is about 13.5 km long, has a maximum width of 1.4 km, and a water surface area of approximately 10 km^2^. The mean depth is about 2.5 m, with a maximum depth of 5 m. A narrow 2.5‐km narrow channel sometimes links the lake to the sea when catchment flooding occurs or under surge conditions associated of extreme high tides (Sinclair, Lane, & Grindley, 1986). The salinity of water in the lake declines from mesohaline in the lower vlei to oligohaline toward the head of the system (Robertson, 1980).

The most recent increase in sea level during the Flandrian Transgression 12,000–4,000 years BP would have flooded the Verloren Valley (Rogers, 1980). When the sea level receded from its Flandrian peak about 4,000 years ago to its present level, the formerly large estuary mouth would have changed into a narrow inlet. This was because large barrier dunes developed along the coast, pushing the narrowing mouth channel southwards so that it now choked between the barrier dunes in the north and rocks in the south (Fromme, 1985).

The confirmed indigenous fish assemblage of Verlorenvlei comprises all three indigenous freshwater species present in the inflowing river, namely *Sandelia capensis* (Anabantidae), *Galaxias zebratus* (Galaxiidae), and *Pseudobarbus verloreni* (Cyprinidae); as well as at least four marine and estuarine species, namely *Lithognathus lithognathus* (Sparidae), *Liza richardsonii* (Mugilidae), *Mugil cephalus* (Mugilidae), and *G. aestuaria* (Clupeidae).

As was the case in other temperate coastal lakes and lagoons, alien freshwater fish species were introduced to this system from the mid‐1900s onwards. These included the centrarchids *Micropterus dolomieu* and *Micropterus salmoides*, the cyprinids *Tinca tinca* and *Cyprinus carpio*, and the cichlid *Oreochromis mossambicus*. The latter two species now dominate the ichthyofauna of Verlorenvlei from a biomass perspective (Sinclair et al., 1986). The banded tilapia *Tilapia sparrmanii* has also been sampled from Verlorenvlei but, due to its inability to tolerate saline waters, it probably only occurs close to freshwater inputs to the lake.

### 2.5 De Hoop Vlei

This brackish coastal lagoon is situated in the De Hoop Provincial Nature Reserve (34°26′S; 20°22′E) and is approximately 4–11 m above MSL. The lagoon is about 18 km long and 0.5 km wide, having a water surface area of approximately 750 ha when full. Water depth and salinity is variable, depending on freshwater flooding of the area, particularly from the Sout and Potteberg rivers. Following extensive flooding, an area of up to 3,000 ha on the plain southwest of De Hoop Vlei may be inundated to a depth of 3 m (Butcher, 1984). During such periods, the maximum water depth of the resulting coastal lake is 7 m and the salinity 3. Conversely, during prolonged droughts, the system is known to almost dry out, thus decimating fish populations in the lagoon. Salinity/conductivity is not uniform across the system and salinity levels ranging between 2 and 15 have been recorded for De Hoop Vlei at three different sites (Day, 1993). The ionic content of the vlei waters is reflective of the proportions found in the sea.

De Hoop Vlei would have been linked to the marine environment during earlier Holocene times and was therefore a fully functional estuary before becoming isolated by 2.5 km of mobile dunefields that run parallel to the coast and are now significantly higher in altitude than the vlei (Lanz, 1997). A full array of marine and estuarine fish species would have been prevalent within the De Hoop estuarine lagoon but after the cessation in connectivity with the sea, all trapped marine species would have become locally extinct once they had died of old age, leaving behind only a few relict estuarine taxa that could conduct their entire life cycle within the system (e.g., *A. breviceps* and *G. aestuaria*). These resident species are likely to have disappeared when the coastal lagoon dried up during subsequent prolonged droughts, and this would account for their apparent absence from the lagoon today.

It is of interest to note that the nearby Soetendalsvlei (34°42′S; 19°59′E), a lagoon that is intermittently linked to the downstream Heuningnes Estuary by a narrow sinuous channel, is known to support sparid (e.g., *Lithognathus lithognathus*) and mugilid (e.g., *Mugil cephalus*) fish populations, with both families requiring some marine or estuarine connectivity in order to be present in the system. Any loss in the connection with the above shallow channel would result in these estuary‐associated marine species disappearing from Soetendalsvlei within a period of one or two decades.

Following isolation, the freshwater fish *S. capensis* would have been able to colonize the De Hoop Vlei from the inflowing rivers at any time when salinities became oligohaline. At present, this species is the only indigenous fish recorded in the lagoon (Scott & Hamman, 1988) but it is probable that the brackish water tolerant *Galaxias zebratus* also colonizes the system from the same river catchments when salinities in the vlei are suitable.

Non‐native fish introductions between 1947 and 1948 included a total of 40 *Lepomis macrochirus*, 440 rainbow trout *Oncorhynchus mykiss* (Salmonidae), 950 *Micropterus dolomieu*, 1,540 spotted bass *Micropterus punctulatus*, and 2,350 *Micropterus salmoides* but none of these non‐native species established within the system (van Rensburg, 1966). The freshwater *Oreochromis mossambicus* was then introduced into De Hoop Vlei in 1958. This species thrived under the variable salinity conditions, exhibiting a growth rate that exceeded that of this species elsewhere in South Africa (van Rensburg, 1966). Consequently, the current fish assemblage at De Hoop Vlei is made up almost entirely of *O. mossambicus* as other freshwater fish species have a limited tolerance to the mesohaline salinities that often prevail within this system.

### 2.6 Wilderness lakes (Rondevlei and Langvlei)

These two estuarine lagoons are located in the upper sections of the Wilderness lakes system on the southern coast of South Africa. The narrow and shallow linkages between these lagoons and the rest of the Wilderness system have been artificially maintained in recent decades to facilitate connectivity (Russell, 2003). Rondevlei (33°59′S; 22°42′E) is round in shape and has a water surface area of 1.43 km^2^, whereas Langvlei (33°59′S; 22°40′E) is more elongated and has a surface area of 2.16 km^2^. The median salinity of Rondevlei between 1990 and 2010 has ranged from 15 in 1991 to 6 in 2008, whereas that of Langvlei has ranged from 11 to 4 over the same period (Russell, 2013).

According to Martin (1962), both Langvlei and neighboring Rondevlei are not associated with a river drainage system and therefore do not arise from a drowned river valley, as did the nearby Swartvlei system. The former two systems may have been the result of marine flooding of large deflation basins during the Flandrian Transgression (Hill, 1975). Subsequent infilling and segmentation between the lakes has occurred due to more recent sediment movement and deposition, such that Rondevlei now has a maximum depth of approximately 6 m and Langvlei a maximum depth of 4 m (Hall, Whitfield, & Allanson, 1987). The narrow and shallow connections between Rondevlei and Langvlei, and between Langvlei and Eilandvlei, threatened to close completely during the second half of the 1900s and were artificially cleared of encroaching vegetation and deepened in an attempt to facilitate continued connectivity, albeit on a reduced scale when compared to earlier times.

Despite the artificial maintenance and improvement of the linkages between Rondevlei and Langvlei, and between Langvlei and Eilandvlei, a number of estuary‐associated marine fish species that occur in Eilandvlei and the adjacent Touw Estuary do not presently occur in either Langvlei or Rondevlei, despite the higher and more favorable salinities in the latter two lagoons (Table 1). Thus, only 21 fish species, including four introduced non‐native freshwater species (mosquitofish *Gambusia affinis*,* Micropterus salmoides*,* Cyprinus carpio* and *Oreochromis mossambicus*), occur in Langvlei/Rondevlei, whereas 32 species occur in the Eilandvlei/Touw portion of the system, including the same four alien freshwater taxa (Table 1). In the event of complete closure of these connections as part of the natural succession taking place in the Wilderness system, the marine fish species from both upper lagoons would disappear and the ichthyofaunal diversity of Rondevlei and Langvlei would be limited to relict estuarine and introduced alien freshwater species.

**Table 1:** Fish species recorded in the upper Wilderness system (Rondevlei and Langvlei) compared to those recorded in the lower Wilderness system (Eilandvlei and Touw Estuary) (data from Hall et al., 1987; Olds et al., 2011; Olds, James, Smith, & Weyl, 2016)

Fish family	Fish species	Rondevlei and Langvlei	Eilandvlei and Touw Estuary
**Freshwater guild**			
Cichlidae	Oreochromis mossambicus*	x	x
Centrarchidae	Micropterus salmoides*	x	x
Cyprinidae	Cyprinus carpio*	x	x
Poeciliidae	Gambusia affinis*	x	x
**Diadromous guild**			
Anguillidae	*Anguilla mossambica*	x	x
**Estuarine guild**			
Atherinidae	*Atherina breviceps*	x	x
Clinidae	*Clinus superciliosus*	x	
Clupeidae	*Gilchristella aestuaria*	x	x
Gobiidae	*Caffrogobius gilchristi*		x
	*Psammogobius knysnaensis*	x	x
	*Redigobius dewaali*		x
Hyporhamphidae	*Hyporhamphus capensis*	x	x
Syngnathidae	*Syngnathus temmincki*	x	x
**Marine guild**			
Ariidae	*Galeichthys feliceps*		x
Carangidae	*Lichia amia*	x	x
	*Trachurus capensis*		x
Elopidae	*Elops machnata*		x
Haemulidae	*Pomadasys commersonnii*	x	x
Monodactylidae	*Monodactylus falciformis*	x	x
Mugilidae	*Liza richardsonii*	x	x
	*Liza dumerili*	x	x
	*Liza tricuspidens*	x	x
	*Mugil cephalus*	x	x
	*Myxus capensis*	x	x
Sciaenidae	*Argyrosomus japonicus*		x
Soleidae	*Heteromycteris capensis*		x
	*Solea turbynei*		x
Sparidae	*Diplodus capensis*		x
	*Rhabdosargus holubi*	x	x
	*Rhabdosargus sarba*		x
	*Lithognathus lithognathus*	x	x
	*Sarpa salpa*		x
Tetraodontidae	*Amblyrhynchotes honckenii*		x

Life histories of the fish species are categorized according to the guilds proposed by Potter, Tweedley, Elliott, and Whitfield (2015), with introduced alien fishes being indicated by an asterisk (*).

## 3 SUBTROPICAL COASTAL LAKES/LAGOONS

### 3.1 Cubhu and Mzingazi

Lakes Cubhu (28°51′S; 31°58′E) and Mzingazi (28°46′S; 32°06′E) are coastal lakes at Richards Bay on the north coast of KwaZulu‐Natal that have been artificially isolated from their marine connection by the construction of the Port at Richards Bay. Both lakes are fed by small, clear coastal streams with substrates comprised predominantly of sandy sediments. Cubhu has a catchment area of approximately 80.2 km^2^ (Cyrus, Wellmann, & Martin, 1993) while that of Lake Mzingazi is 164 km^2^ (Weerts, MacKay, & Cyrus, 2014). Lake water surface areas are approximately 460 and 1,100 ha, respectively. Both systems have maximum depths below sea level, with Cubhu being approximately −4 m (Martin & Cyrus, 1994) and Mzingazi −14 m (Kelbe, Germishuyse, & Fourie, 2001).

Under present‐day conditions, both lakes are freshwater. Salinity is likely to have penetrated into these systems in the early Holocene and would certainly have performed so during the mid‐Holocene sea level highstand of 3.5 m above present sea level. During this time, both lakes would have functioned as estuarine arms of a greater estuary which covered the present‐day Mhlathuze Estuary and the Richards Bay harbor region. Fishes present would have comprised an array of marine and estuarine species, probably similar to that typical of permanently open estuaries on the present‐day KwaZulu‐Natal coast.

Sea level retreat to approximately 2 m below present levels 3,000 years ago (Figure 3; Ramsay, 1995) would have seen both lakes become freshwater systems. During this period, estuarine resident and freshwater fishes would have been prevalent and marine species would have been lost from both lakes. A subsequent rise in sea level to present levels would have seen closer proximity between saline estuarine waters and these lakes, but conditions in the lakes would have remained predominantly fresh. Saline intrusion would have been likely during drought conditions when lake water levels dropped. During this period, estuarine resident and freshwater fishes (e.g., tilapia *Oreochromis mossambicus*) would have remained dominant but a host of marine fishes would have recruited into the lakes and used these habitats as nursery areas. These fishes would have included species that are, under present‐day conditions, common in Lake Nhlange in the linked Kosi Bay lakes system to the north. Marine taxa would have included the tarpon (*Megalops cyprinoides*), several grey mullet species (e.g., *Myxus capensis, Mugil cephalus* and *Liza alata*), moonies (*Monodactylus* spp.), pursemouths (*Gerres* spp.), and silverbream (*Rhabdosargus* spp.). Estuarine resident species would have been dominated by *G. aestuaria*,* A. breviceps*,* Hyporhamphus capensis*, and *Ambassis* spp.

Recent history has seen significant changes in the greater Richards Bay. Port development in the 1970s spilt Richards Bay Lagoon into two separate estuarine areas (Mhlathuze Estuary and the Port of Richards Bay) and both coastal lakes have had outlet points dammed and raised over the last 70+ years, mainly to maximize abstraction yields (Weerts & Cyrus, 2001). While outlet weirs have significantly increased volumes of these coastal lakes, their waters retain relatively high levels of conductivity which is reflective of their geophysical origins. This possibly plays a role in having allowed select estuarine macrobenthic taxa (isopods and amphipods) to maintain good populations in both systems (Mackay & Cyrus, 2001). Outlet weirs have, however, served as barriers to natural migrations of fauna between the freshwater lake habitats and downstream estuarine and marine habitats. This has impacted local populations of species, with fishes being especially affected (Weerts et al., 2014).

Both lakes retain a compliment of freshwater fishes typical of KwaZulu‐Natal coastal freshwaters, with 11 species of freshwater fish having been recorded in Lake Cubhu, and 10 reported from Lake Mzingazi (Weerts and Cyrus, 2001). Fish communities are dominated by cichlids, predominantly *O. mossambicus* (which is native to the region) and *Coptodon rendalli*; with *Clarias gariepinus* (Clariidae) and several small *Aplocheilichthys* (Poecilidae) and *Enteromius* species (Cyprinidae) also common. No non‐native fishes have been reported from either system, although *Coptodon rendalli* may have been translocated.

Both lakes retain some estuarine species (three species in Cubhu, four in Mzingazi) with *G. aestuaria* dominating. As a result of some connectivity to the marine environment, anguillid eels occur in both lakes. As obligate catadromous species, they are capable of overcoming significant barriers to complete their natural recruitment migrations as elvers into freshwater habitats. The abundance of marine fishes in both lakes has waned with the development of weirs and a consequent lack of new recruits. This has happened to varying degrees in the lakes, with sampling in 2009 (S. Weerts, unpublished data) showing that *M. capensis* still manages to recruit into the Lake Cubhu via an outflow that cascades over a relatively gentle gradient. *Acanthopagrus vagus*,* Elops machnata,* and *Monodactylus falciformis* have also been sampled in the lake postweir installation in 1987 (Weerts & Cyrus, 2001).

Sampling in Lake Mzingazi during 2001 and 2002 (Weerts et al., 2014) and 2009 (S. Weerts, unpublished data) returned no marine fishes because the vertical drop outflow completely prevents their recruitment from the estuary. Marine fishes that had been reported as being present in the system during 1985 were preweir installation but those captured in 1993 (postweir installation) are likely to have been recruited during extreme flood events when a natural drainage line around the weir was activated (Weerts et al., 2014).

### 3.2 Lake Sibaya

Lake Sibaya (27°21′S; 32°41′E) is a coastal lake approximately 60–77 km^2^ in area, an average depth of 13 m, and a water surface about 20 m above mean sea level (Hill, 1979). The maximum depth in the lake is about 40 m, thus making the deepest point about 20 m below MSL. The conductivity of water in the lake, based on 44 samples collected between 1969 and 1982, was 0.6 mS/cm (Day, 1993), with marginally elevated concentrations of various ions in its “fresh” water (Allanson & van Wyk, 1969).

Well‐developed offshore submarine canyons are indicative of the Phongolo River having flowed in an easterly direction through the Sibaya basin area during the Pleistocene, thus creating a large estuary in the vicinity of the current lake when sea levels rose (Maud, 1968). However, the redirection of the Phongolo River toward the north meant that the Sibaya estuarine system had no river flow to maintain a permanently open mouth which was closed by littoral aeolian processes (Hobday, 1979). According to Wright, Miller, and Cooper (2000), estuarine lagoonal conditions at Sibaya ceased approximately 5,000 years BP when barrier dune formations impounded the previous estuary mouth region. Sibaya is estimated to have had an initial water surface area almost 150 km^2^ prior to sediment infilling during the latter part of the Holocene (Orme, 1973), that is, the lake was originally about double the current surface area.

Once vegetation had colonized and stabilized the resulting coastal dunes, the marine and estuarine fauna would then have been permanently isolated in the newly formed coastal lake (Hill, 1975). The marine biota would have died out soon after the loss in connectivity with the sea but remnants of the estuarine invertebrate and fish fauna that were able to reproduce under freshwater conditions remained within the lake (Allanson et al., 1966).

The ichthyofauna of the original Sibaya Estuary would have had a species diversity resembling that of the nearby Kosi Estuary to the north (Blaber & Cyrus, 1981), implying that more than 100 species were likely occupying that system when it was fully estuarine. Once the estuary mouth had remained permanently closed for at least 50 years, only five euryhaline estuarine species persisted, namely *A. breviceps*,* G. aestuaria*,* Croilia mossambicus*,* Glossogobius giuris,* and *Silouettea sibayi* (Bruton, 1980a). The remaining 12 freshwater fish species subsequently colonized the coastal lake from the inflowing streams and surrounding freshwater pans once salinities in the system became oligohaline. *Micropterus salmoides* were introduced into the lake in 1935 to provide a sport fishery (Harrison, 1936) but this introduction appears to have failed.

### 3.3 Mgobozeleni

Mgobozeleni (27°31′S; 32°39′E) is a 1.5‐km‐long, 1‐km‐wide lagoon that has a surface area of 1.8 km^2^. Over one‐third of the lake floor is deeper than 3 m, with a maximum depth of 5 m being recorded (Bruton & Appleton, 1975). This lagoon is linked to the sea by a perennial overflow stream that meanders through a swamp forest and reed swamp before entering a mangrove forest that is immediately upstream of the small Mgobozeleni Estuary. The water in the lake is fresh (300 μS/cm), has slightly elevated chloride levels (106 mg/L), and is stained dark brown due to humates from the surrounding vegetation (Bruton & Appleton, 1975).

Mgobozeleni would have been a much larger estuarine lake during the Flandrian Transgression. Following the retreat in sea level, the lake decreased from about 40 km^2^ during the Holocene to <2 km^2^ at present, a decline in water surface area of more than 90% (Orme, 1973). While functioning as an estuarine lake, Mgobozeleni would have had a rich diversity of marine and estuarine fish species but these would have progressively declined as the system decreased in size and became increasingly isolated from the marine and estuarine environments. At the same time, declining salinities would have provided an opportunity for freshwater fish species to naturally colonize the lake, especially when salinities became oligohaline or even fresh.

The above scenario is partially reflected in the present‐day fish composition of this system (Table 2) which shows that the very small estuarine portion of the system has a high diversity of 24 marine, eight estuarine, six freshwater, and one diadromous species (total = 39 fish taxa). In contrast, the almost isolated Mgobozeleni Lagoon has 10 freshwater, three diadromous, three estuarine, and two marine species (total = 18 fish taxa). In the event of complete loss of connectivity between the lagoon and sea, Mgobozeleni would lose all diadromous and marine taxa once the present cohorts of these species in the system had perished.

**Table 2:** Fish species recorded in the Mgobozeleni Lagoon (including the adjacent swamp forest and reed swamp) compared to those recorded in the linked Mgobozeleni Estuary (including the adjacent mangrove forest; modified from Table 3 in Bruton, 1980b)

Fish family	Fish species	Lagoon	Estuary
**Freshwater guild**			
Centrarchidae	Micropterus salmoides*	x	
Cichlidae	*Oreochromis mossambicus*	x	x
	*Oreochromis placidus*	x	x
	*Pseudocrenilabrus philander*	x	x
	*Serranochromis meridianus*	x	
	*Coptodon rendalli*	x	
	*Tilapia sparrmanii*	x	x
Clariidae	*Clarias gariepinus*	x	x
Cyprinidae	*Enteromius paludinosus*	x	
	*Enteromius radiatus*	x	
Poeciliidae	*Aplocheilichthys katangae*	x	x
**Diadromous guild**			
Anguillidae	*Anguilla bengalensis*	x	
	*Anguilla bicolor*	x	
	*Anguilla mossambica*	x	x
Estuarine guild			
Clupeidae	*Gilchristella aestuaria*		x
Atherinidae	*Atherina breviceps*		x
Syngnathidae	*Microphis brachyurus*		x
Ambassidae	*Ambassis dussumieri*		x
	*Ambassis natalensis*		x
Gobiidae	*Awaous aenofuscus*	x	x
	*Glossogobius giuris*	x	x
Eleotridae	*Eleotris melanosoma*	x	x
**Marine guild**			
Carangidae	*Caranx sexfasciatus*		x
	*Trachynotus botla*		x
Haemulidae	*Plectorhinchus gibbosus*		x
	*Pomadasys commersonnii*		x
Kuhlidae	*Kuhlia rupestris*		x
Lutjanidae	*Lutjanus argentimaculatus*		x
Megalopidae	*Megalops cyprinoides*	x	
Monodactylidae	*Monodactylus argenteus*		x
Mugilidae	*Crenimugil crenilabis*		x
	*Liza alata*		x
	*Liza dumerili*		x
	*Liza macrolepis*	x	x
	*Liza tricuspidens*		x
	*Mugil cephalus*		x
	*Myxus capensis*		x
	*Valamugil buchanani*		x
	*Valamugil cunnesius*		x
	*Valamugil robustus*		x
	*Valamugil seheli*		x
Polynemidae	*Polydactylus plebius*		x
Sparidae	*Acanthopagrus vagus*		x
	*Diplodus capensis*		x
	*Rhabdosargus sarba*		x
	*Rhabdosargus thorpei*		x
Teraponidae	*Terapon jarbua*		x

Life histories of the fish species are categorized according to the guilds proposed by Potter et al. (2015).

*indicates an introduced alien fish species.

In Bhangazi (28°07′36″S; 32°32′22″E), a small freshwater lake/lagoon situated about 70 km south of Mgobozeleni in the vicinity of Lake St Lucia, the only marine fish species recorded from this system is the tarpon *Megalops cyprinoides* (Bruton & Taylor, 1979). The postlarvae of this species are able to negotiate a shallow channel linking Bhangazi to Lake St Lucia during high rainfall years when lake water levels are high. However, the adults appear unable to escape Lake Bhangazi to the sea via St Lucia once they have matured due to the shallow and intermittent nature of the connecting channel.

## 4 DISCUSSION

Historical connectivity with the marine environment and past estuarine conditions in coastal lakes and lagoons is a primary determinant of present‐day fish assemblages. A small and remarkably consistent group of estuarine species have persisted in these coastal lakes. These fishes are all small, estuarine resident species that are capable of completing their life cycles in estuaries and isolated coastal lakes. The pelagic *G. aestuaria* and *A. breviceps* inhabit coastal lakes across the full biogeographic range under consideration here and in the subtropical lakes of northern KwaZulu‐Natal some members of the families Gobiidae and Eleotridae species have also persisted (*Croilia mossambicus*,* Glossogobius giuris*,* Silhouettea sibayi*,* Eleotris* spp.). These are all species with tropical affinities, and their presence in these systems is as a result of biogeographical factors rather than differences in the origin and evolution of coastal lakes along the South African coast. This hypothesis is supported by the successful establishment of *O. mossambicus* in temperate lakes. This cichlid occurs naturally in east flowing rivers down to the Bushman's River in the Eastern Cape Province and is present in the lower reaches of almost all rivers and estuaries within its native range. Upon introduction, this species readily established in the temperate coastal lakes where it is now ubiquitous.

A euryhaline physiology, together with a reproductive style suited to establishment in lentic environments, is important to the success of these estuarine fishes in coastal lakes. A study on the effect of propagule pressure on the establishment success of fishes in irrigation ponds in the Sundays River floodplain by Woodford, Hui, Richardson, and Weyl (2013) demonstrated that the most successful native colonizers were *G. aestuaria*,* Oreochromis mossambicus,* and *Glossogobius callidus*. It is interesting to note that these three species are also well represented in the coastal lake ichthyofauna. Apart from the water chemistry, habitats in these coastal lakes closely resemble that of estuarine lakes in various ways, for example, the presence of submerged aquatic vegetation which is important for egg attachment by species such as *A. breviceps* (Neira, Beckley, & Whitfield, 1988).

Estuarine spawning fishes that are absent from coastal lakes in South Africa include *Ambassis ambassis*, a species that is common in the freshwater reaches of South African subtropical temporarily open/closed estuaries (e.g., Mhlanga Estuary) and estuarine lakes (e.g., Lake Amanzimnyama) but these fishes clearly require intermittent connection with the estuarine environment for long‐term survival in freshwater systems. The loss of connectivity with estuarine systems has also impacted the diversity of marine fishes in all coastal lakes. No marine fishes occur where connectivity has been completely lost (e.g., Sibaya, Groenvlei). The extinction of marine fishes in these systems would have occurred gradually as their linkages with marine and estuarine environments became increasingly constrained over time and salinity regimes shifted from estuarine to fresh. While most estuary‐associated marine fishes in South Africa are tolerant of low salinities rather than hypersaline conditions (Whitfield, Blaber, & Cyrus, 1981) relatively few inhabit completely freshwater as a matter of preference.

In systems that have retained tenuous linkages with the sea (Verlorenvlei, Wilderness lakes, Cubhu), elements of the marine fish assemblage have persisted. Other marine spawning species in “transitioning” estuarine lakes include known facultative catadromous species, such as *M. capensis* in the warm temperate region and *Megalops cyprinoides* in the subtropical region. Additional marine species that typically occur in transitioning estuarine lakes include species that penetrate rivers elsewhere in South Africa (e.g., *Mugil cephalus*,* Acanthopagrus vagus*, and *Monodactylus falciformis*). The numbers and types of marine species occurring in different systems depend upon the nature of the connection with the marine environment and the prevailing salinity regime. Thus, in Rondevlei and Langevlei where connectivity with the greater Wilderness lakes system has been retained and salinities are mesohaline, 16 marine and estuarine species occur, whereas in Verlorenvlei where connectivity is only established under flood conditions and salinities are lower, only three marine species occur. Marine fishes are a consistent component of the fish fauna of Lake Cubhu but in Lake Mzingazi, they occur only irregularly and in low abundance as recruitment opportunity has been limited to sporadic extreme flood events.

The Kosi segmented estuarine lakes system in northern KwaZulu‐Natal supports a fish fauna that is spatially distributed in a manner that reinforces our observations of fish assemblages in evolving lakes and lagoons elsewhere in South Africa and illustrates the interplay of marine/estuarine connectivity and salinity effects. The mouth of Kosi Estuary, with near marine salinities, supports a rich diversity of marine fishes (Blaber, 1978). Most of these do not penetrate the system beyond the estuary and species richness falls away quickly in the next two upstream estuarine lakes (Makhawulani and Mpungwini) which have polyhaline salinities. The fish here are dominated by estuarine‐associated marine and estuarine resident species. Lake Nhlange, 12 km from the mouth and separated from the lower estuarine lakes by a long and narrow channel, has a fresh to oligohaline salinity regime and is dominated by estuarine resident fishes. Marine fishes still occur in good abundances, and these are typically facultative catadromes that penetrate rivers elsewhere in South Africa. Lake Amanzimnyama at the head of the system (20 km from the sea) is fresh, with the estuarine residents *G. aestuaria* and *Ambassis ambassis* dominating fish abundance (S. Weerts, unpublished data). Marine species still occur, but these are rare and restricted to a select few catadromous taxa. Essentially the marine species are replaced by at least nine freshwater species which are the same as those that typically occur in other KwaZulu‐Natal coastal lakes. Thus, in one coastal system, there is strong evidence of the combined effects of changing salinity and connectivity on fish composition as the compartments become increasingly freshwater dominated and less connected with the marine environment.

Freshwater fish diversity in coastal lakes and lagoons is a function of historical and present biogeography and salinity. From a freshwater biogeography perspective, the inflowing rivers of the four temperate systems reviewed here contain three or fewer native freshwater fishes (see Ellender et al., 2014), while the subtropical lakes are fed by river systems that can contain more than 40 freshwater fish species and are periodically connected to one another during times of flood (Thieme et al., 2005). As a result of a low tolerance to salinity, few freshwater fishes penetrate estuaries in southern Africa (Whitfield, 2015). Those that do include *Oreochromis mossambicus*,* Coptodon rendalli*,* Pseudocrenilabrus philander*,* Enteromeus trimaculatus*,* Enteromeus paludinosus,* and *Clarias gariepinus,* and all of these fishes occur in KwaZulu‐Natal coastal lakes within their natural biogeographical range.

Other freshwater species that occur in coastal lakes are invariably secondary freshwater fishes which have some tolerance of brackish water. As is characteristic for the Cape Fold Ecoregion (Ellender & Weyl, 2014, Ellender et al., 2017), rivers feeding Verlorenvlei, De Hoop Vlei, the Wilderness lakes, and Groenvlei contain few native species. This, in conjunction with the relatively high salinities in some of these systems, explains the low representation of native freshwater fishes. The Verloren River, for example, contains only the three native species that are represented in the lake. Elevated salinity is also a likely key factor in the occurrence and distribution of freshwater fishes in De Hoop Vlei. Despite being connected to a relatively large catchment of approximately 1,200 km^2^, only *Sandelia capensis* occurs in the lake and only during oligohaline phases (Harrison, 1957). In the Wilderness Lakes, the inflowing river Duiwe River contains the barb *Pseudobarbus swartzi* but salinities of up to 8 are likely to the primary factor preventing its occurrence in the lakes.

**Figure 5:**
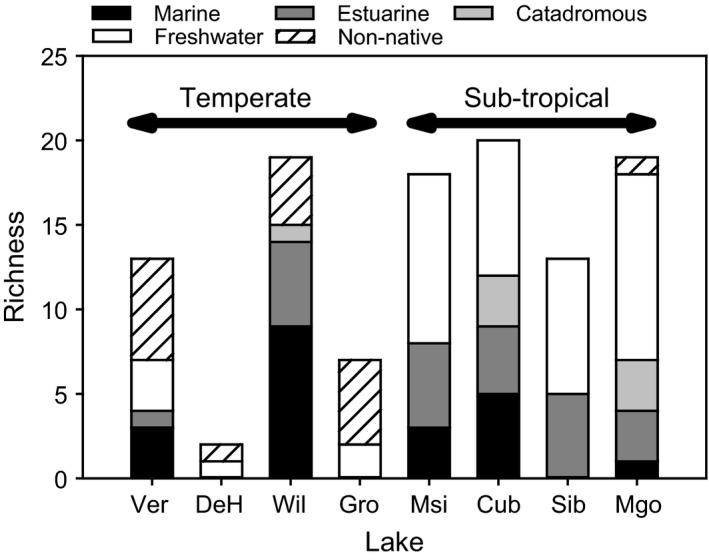
Fish species richness in four temperate (Ver, Verlorenvlei; DeH, De Hoop Vlei; Wil, Wilderness; Gro, Groenvlei) and four subtropical (Msi, Mzingazi; Cub, Cubhu; Sib, Sibaya; Mgo, Mgobozeleni) coastal lakes/lagoons based on fish guild composition in each of the systems.

Subtropical coastal lakes in KwaZulu‐Natal all have freshwater conditions or very low salinities (<1) and support a more diverse freshwater fish assemblages (10–12 species) compared to temperate coastal lakes (Figure 5, Table 3). Historical connectivity to larger catchments may play a role in all three subtropical case studies. Lakes Cubhu and Mzingazi were both part of a larger Richards Bay system that was linked to the Mhlathuze River catchment, and Lake Sibaya was previously connected to the large Phongola River. Biogeographical considerations, however, are a more proximate reason why coastal lakes in KwaZulu‐Natal support higher freshwater fish species diversity than do similar systems in temperate regions (Weyl & Cowley, 2015). In both zoogeographical regions *A. breviceps* and *G. aestuaria* feature as relict species capable of occupation of both estuarine and coastal lakes and lagoons. Although other estuarine spawning species such as *Croilia mossambicus* and *Glossogobius giuris* are also capable of making the transition during the evolutionary changes in these aquatic ecosystems, many marine and some estuarine taxa appear to rely on continued connectivity with the sea in order to persist within such water bodies.

**Table 3:** Summary of fish species occurrence in eight coastal lakes and lagoons

Fish family	Fish species	Ve	DH	LR	Gr	Mz	Cu	Si	Mg
**Freshwater guild**									
Anabantidae	*Ctenopoma multispine*							x	
	*Sandelia capensis*	x	x						
Cichlidae	*Oreochromis mossambicus*	x	x	x	x	x	x	x	x
	*Oreochromis placidus*								x
	*Pseudocrenilabrus philander*					x	x	x	x
	*Serranochromis meridianus*								x
	*Coptodon rendalli*					x	x	x	x
	*Tilapia sparrmanii*	x				x	x	x	x
Centrarchidae	Micropterus salmoides*	x		x	x				x
	Micropterus dolomieu*	x							
	Lepomis macrochirus*				x				
Clariidae	*Clarias gariepinus*					x	x	x	x
	*Clarias theodorae*						x	x	
Cyprinidae	*Enteromius paludinosus*					x		x	x
	*Enteromius radiatus*								x
	*Enteromius viviparous*					x	x		
	*Labeo molybdinus*							x	
	*Pseudobarbus verloreni*	x							
	Cyprinus carpio*	x		x	x				
	Tinca tinca*	x							
Galaxiidae	*Galaxias zebratus*	x							
Mormyridae	*Marcusenius macrolepidotus*					x	x	x	
Poeciliidae	*Aplocheilichthys katangae*					x			x
	*Aplocheilichthys johnstonii*					x			
	*Aplocheilichthys myopasae*							x	
	Gambusia affinis*				x				
**Catadromous guild**									
Anguillidae	*Anguilla bengalensis*								x
	*Anguilla bicolor*						x		x
	*Anguilla mossambica*			x			x		x
	*Anguilla marmorata*						x		
**Estuarine guild**									
Atherinidae	*Atherina breviceps*			x	x			x	
Clinidae	*Clinus superciliosus*			x					
Clupeidae	*Gilchristella aestuaria*	x		x	x	x	x	x	
Eleotridae	*Eleotris melanosome*								x
	*Eliotris fuscus*						x		
Gobiidae	*Awaous aenofuscus*								x
	*Glossogobius callidus*					x			
	*Glossogobius giuris*					x	x	x	x
	*Croilia mossambicus*					x	x	x	
	*Psammogobius knysnaensis*			x					
	*Silouettea sibayi*							x	
Hyporhamphidae	*Hyporhamphus capensis*			x		x			
Syngnathidae	*Microphis brachyurus*			x					
**Marine guild**									
Carangidae	*Caranx sexfasciatus*					x			
	*Lichia amia*			x					
Haemulidae	*Pomadasys commersonnii*			x					
Megalopidae	*Megalops cyprinoides*					x	x		x
	*Elops machnata*						x		
Monodactylidae	*Monodactylus falciformis*			x			x		
Mugilidae	*Liza richardsonii*	x		x					
	*Liza dumerili*			x					
	*Liza macrolepis*								x
	*Liza tricuspidens*			x					
	*Mugil cephalus*	x		x					
	*Myxus capensis*			x		x	x		
	*Acanthopagrus vagus*						x		
	*Rhabdosargus holubi*			x					
Sparidae	*Lithognathus lithognathus*	x		x					

Ve, Velorenvlei; DH, De Hoop Vlei; LR, Langvlei and Rondevlei; Gr, Groenvlei; Mz, Mzingazi; Cu, Cubhu; Si, Sibaya; Mg, Mgobezeleni.

Fish species guilds are categorized according to Potter et al. (2015) (*alien species). Note that *Oreochromis mossambicus* is introduced into the temperate coastal systems but native in subtropical areas.

Recent history has seen significant changes in the freshwater fish fauna of South African coastal lakes, especially in the Cape systems which naturally support a very limited diversity of these fishes. These have been caused by translocations of subcontinental fishes beyond their natural distribution limits and the introduction of alien species. The main driver for these introductions has been the enhancement of fisheries for angling (Ellender & Weyl, 2014). Some non‐native fishes have persisted in most temperate lakes but they are generally absent from the subtropical systems. The reasons for this are unclear, but as there is evidence of the failed introduction of at least one species into Lake Sibaya, biotic resistance from the richer native fish communities is a possibility.

The impacts of these introductions and translocations have not been studied in coastal lakes in South Africa. In these closed systems, it is probable that predation impacts on small estuarine fishes are significant. Predatory fishes have been observed feeding on native estuarine fishes (e.g., Weyl & Lewis, 2006) but *A. breviceps* and *G. aestuaria* seem to have persisted in the presence of alien fishes for more than 80 years in Groenvlei. As alien fish have been linked to impacts at all levels of organization in freshwater ecosystems (see Ellender & Weyl, 2014 for a review), research into the impacts of these species on indigenous fish assemblages in coastal lake ecosystems is urgently required.

The historical changes in the fish faunas of coastal lakes are examples of just one of the growing threats to these systems that are indicative of changes that have been introduced during a new epoch, the “Anthropocene,” when human activities have become a significant impact on the earth's geology, atmosphere, and ecosystems (Crutzen & Stoermer, 2000). While the onset of this epoch is debated, the Industrial Revolution (1800) has been proposed as a logical starting point (Steffen, Grinevald, Crutzen, & McNeill, 2011). Impacts to South African coastal lakes have become increasingly significant over the last 100 years, when agricultural (including forestry) development in the catchments of these systems has grown, with concomitant reductions in ground water levels and surface water inflows to coastal systems. This has affected salinity regimes in these systems and has likely impacted both the flora and fauna of these lakes.

In KwaZulu‐Natal, direct abstraction of freshwater occurs for municipal, industrial, agricultural, and mining use. Forestry in the catchments of Lake Sibaya has exacerbated the impact of prolonged drought, and in 2016, the lake was lower than ever previously recorded, to the extent that the system was divided into two basins separated by a dry sand spit. The installation of weirs in the coastal lakes in the vicinity of Richards Bay to increase abstraction yields has caused barriers to fish migration and significantly impacted on the ability of marine fish faunas to enter or leave these lakes.

In the Western Cape Province, the introduction of alien and translocated fishes has been the overriding cause of changes in coastal lake fish faunas since the retreat of sea levels 3,000–4,000 years ago. It appears that these introductions have not led to localized extinctions of relict estuarine fish populations, but they certainly have placed pressure on them, through predation and/or competition. These isolated populations of estuarine fishes have unique genetic potential and deserve special conservation attention (Phair et al., 2015). The unique nature of these coastal lakes, which are South Africa's only natural lake systems, warrants further research attention. Water abstraction, water pollution, connectivity with other freshwater and estuarine habitats, and the introduction of alien and translocated fishes are key issues which need to be considered in management strategies.

In an international context, the estuarine to coastal lake/lagoon evolutionary processes that occurred in southern Africa have been repeated on coastal plains around the world. In some cases, the resulting conditions in these systems are more extreme than those experienced on the African subcontinent. For example, in south‐western Australia, some systems are normally closed for several years at a time and only open to the sea following episodic floods (Hodgkin & Hesp, 1998). These systems are characterized by a restricted euryhaline estuarine biota that is likely to decline as salinities increase (Sosa‐López, Mouillot, Ramos‐Miranda, Flores‐Hernandez, & Chi, 2007). Those systems that are permanently closed to the sea tend to form salt lakes and become grossly hypersaline and may dry up altogether (Hodgkin & Hesp, 1998), thus resulting in a complete loss of all ichthyofauna under such conditions. In contrast, coastal lagoons and lakes in other parts of the world do not have hypersalinity issues and are characterized by ichthyofaunas that comprise marine, estuarine, and freshwater guilds, with the composition being strongly linked to the magnitude and duration of connectivity with the sea (Pérez‐Ruzafa, Mompeán, & Marcos, 2007).

## CONFLICT OF INTEREST

None declared.
